# How did the COVID-19 pandemic affect food environment, food purchase, and fish consumption among low-income urban households in Bangladesh—A path analysis

**DOI:** 10.3389/fpubh.2022.994236

**Published:** 2022-09-15

**Authors:** Mahsina Syeda Akter, Elise F. Talsma, Edith J. M. Feskens, Shakuntala H. Thilsted, Sabrina Rasheed

**Affiliations:** ^1^Division of Human Nutrition and Health, Wageningen University and Research, Wageningen, Netherlands; ^2^WorldFish, Penang, Malaysia; ^3^Health Systems and Population Studies Division, International Centre for Diarrhoeal Disease Research, Bangladesh (icddr,b), Dhaka, Bangladesh

**Keywords:** food environment, food access, food price, affordability, urban poor, fish consumption, COVID-19 pandemic, informal settlements

## Abstract

**Background:**

Animal source foods, especially fish is the most commonly consumed and an important source of macro and micronutrients in the diet of the urban low-income residents. The COVID-19 pandemic has disrupted the food environment in Bangladesh but little is known about how food access and food prices (affordability) have affected the purchase and consumption of fish. The objective of the study was to understand the impact of the first wave of the COVID-19 pandemic on urban food environment with a specific focus on fish consumption.

**Methods:**

A cross-sectional survey was conducted among 586 homogeneous adults (288 females and 298 males) from separate households from five informal settlements in Dhaka city, Bangladesh during October-November 2020. Data were collected on: (1) food access and affordably; and (2) food purchase and fish consumption. The associations between food access, price, food purchase, and fish consumption were evaluated using path analysis.

**Results:**

The majority of respondents reported that food access was more difficult, food prices increased, and food purchase decreased during the COVID-19 pandemic compared to pre-COVID (84–89% of respondents). Fish and meat were more difficult to access, more expensive and purchased less compared to other foods (74–91% of respondents). Compared to pre-COVID period, households consumed less fish during the COVID-19 pandemic, and reported compromised the variety and quality of fish. In the path analysis, food access was associated with food purchase (*b* = 0.33, *p* < 0.001). Food purchase was associated with quantity, variety, and quality of fish consumed. Food price was inversely associated with the quality of fish consumed (*b* = −0.27, *p* < 0.001).

**Conclusions:**

The COVID-19 pandemic negatively affected the food environment, particularly food access, price (affordability), purchase, and consumption, especially of fish. Limited food access negatively affected the quantity, variety and quality of fish consumed. An increase in food prices directly affected the quality of fish consumed. Policy actions are essential to ensure equal access to nutritious foods, such as fish. These policies need to focus on diversity and quality along with preventing increases in food prices during emergencies to mitigate future threats to the nutrition and health of the urban low-income residents.

## Introduction

Containment measures of the COVID-19 pandemic had a severe negative impact on the livelihood of people, food, and nutrition security and diet quality among low-income populations in low- and middle-income countries (LMICs) including Bangladesh (1, 2). In Bangladesh, the impact of the COVID-19 pandemic was associated with a decline in consumption of fruits and vegetables, and animal source foods, such as fish and meat (3–8). In Bangladesh, fish is the most commonly consumed animal sourced food and an important source of macro-and micronutrients in the diet of the low-income populations (9–11). Consuming different fish species (variety) is important as their nutrient composition vary substantially; for example, farmed species contribute to lesser micronutrient intakes than non-farmed ones (12–14). During the pandemic, in studies conducted among low- and middle-income urban population of Bangladesh, researchers reported that fish consumption reduced (4, 5, 8), with more people eating fish less than five times a week after the beginning of the pandemic than before the pandemic in the low-income group (29 vs. 6%, respectively) (4). The researchers also reported that fewer fish species were available in the market and the price of fish increased during the COVID-19 pandemic (4).

Food consumption and acquisition is shaped by the food environment. The food environment is defined as the interface in which consumers interact with various domains within external (food availability, prices, vendor, product properties and marketing, and regulation policies) and personal dimensions (food accessibility, affordability, convenience, and desirability of foods) (15). The food environment is particularly challenging for the urban low-income population living in informal settlements, as they rely on daily wages for income and informal markets for food (16, 17). At the onset of the COVID-19 pandemic, researchers projected the possible impact on the global food environment and highlighted the vulnerability of the urban low-income residents (18–20). The urban low-income population in informal settlements has become more vulnerable since the beginning of the pandemic in Bangladesh, as they already lived with many challenges before the pandemic, for example, persistent food and nutrition insecurity, poor infrastructure, overcrowding housing, inadequate water supply, inadequate healthcare services and social safety net programs (21–23). Consequently, they suffered from severe food insecurity and faced greater challenges with respect to acquiring food during the COVID-19 pandemic (24, 25). It is, therefore, important to understand the impact of the COVID-19 pandemic on the food environment and its domains, and on food purchase and food consumption in this population in order to prevent them from falling even further behind in achieving the 2030 Sustainable Development Goals (SDGs), ending hunger and access to safe, nutritious and sufficient food for all people; and eradicating all forms of malnutrition (26).

During the COVID-19 pandemic in Bangladesh, in a range of studies with various focuses (such as food security, nutrition, and food systems), researchers demonstrated that disruptions to supply chain, transportation, shorter market opening hours, market displacement, and lowered vendor mobility were associated with limited access to food which adversely affected food purchase and consumption (19, 20, 27). Only one study with a focus on the food environment documented sufferings of consumers from 119 countries including Bangladesh, and reported that among the personal domains of food environment, limited access to food and reduced ability to purchase food (affordability) among low-income population were associated with reduced food purchase and thus consumption (28, 29). The increase in food prices during the COVID-19 pandemic was also associated with lower purchase and consumption behaviors (7, 27, 30). However, to the best of our knowledge, it has not yet been reported how the COVID-19 pandemic affected the food environment among urban low-income consumers in the informal settlements of Bangladesh. Moreover, whether changes in personal domains of the food environment contributed to reduced fish consumption is unknown. In addition, it is not clear which of these food environment domains, i.e., food access and affordability, were most associated with reduced fish consumption.

Therefore, this study aimed to understand changes in the urban food environment (personal dimension) during the first wave of the COVID-19 pandemic, and the impact on fish consumption among low-income households in Dhaka city.

## Materials and methods

### Study design and sample

A cross-sectional household survey through mobile phone was employed to integrate quantitative measures of changes in the food environment components, as well as on food purchase and household fish consumption. The survey was conducted between 10 October and 12 November 2020, during the 1st wave of the COVID-19 pandemic as part of the Urban Health and Demographic Surveillance System (UHDSS) of International Centre for Diarrhoeal Disease Research, Bangladesh (icddr,b). The UHDSS covers 31,577 households in five informal settlements, namely, Korail, Mirpur, Shampur, Dholpur, and Tongi of Dhaka North, Dhaka South and Gazipur city corporations (22). Assuming a population proportion of 50%, a 20% response rate at 5% absolute precision with a 95% level of significance and effect size of 1.5, the estimated sample size was 693 (31). The number of households in each of the five informal settlements was determined using a probability proportional to size (PPS) sampling method in which each household was chosen randomly from a sequence of random numbers obtained from a web-based random number generator. From each household, one adult female or male, aged 18 years or over was interviewed with a semi-structured questionnaire. The questionnaire included sections on household characteristics, impact of COVID-19 on food environment, and mental health characteristics. Information on the age, sex, years of schooling, current occupation of respondents, number of rooms, and number of family members were also asked. We assessed household crowding index (number of people per room). Detailed sampling and data collection procedure are described elsewhere (31).

### Assessing changes in the personal food environment during the COVID-19 pandemic

We collected information on perceived changes in the personal domains of the food environment, particularly accessibility and affordability. The household access to food was assessed by asking “how easy was accessing food during Corona?.” The responses were recorded with four categories: “more difficult than usual,” “easier than usual,” “more difficult for some foods,” and “same as before.” Additional questions with yes/no responses were asked to determine which foods of six food groups: fresh vegetables, fish/meat, dry food items (rice, lentils, spices, etc.), eggs, dairy, and fruit, were difficult to access. Furthermore, the reasons behind the difficulties to access foods were investigated with close ended-questions based on previous literature (32) and the qualitative part of the study.

For affordability, how COVID-19 affected changes in food price during the pandemic was explored with four options for response: “increased more than usual,” “decreased more than usual,” “increased for some foods,” and “remained the same.” If “increased more than usual” was the response, then additional question was asked about which foods were more expensive.

### Assessing changes in household food purchase behavior during the COVID-19 pandemic

Changes in household food purchases during the COVID-19 pandemic were assessed with the response categories “less than usual,” “same as before,” and “more than usual.” If “less than usual” was answered, each respondent was asked additional questions about which foods were most expensive in the six food groups mentioned above, why she/he bought less food, and where she/he bought most of the food.

### Assessing changes in household fish consumption during the COVID-19 pandemic

The question “how did household fish consumption change during Corona?” was asked to assess changes in total quantity of fish consumption. Additional questions on changes in the variety (different species) and quality of household fish consumption were asked separately. Responses were recorded using three categories “less than usual,” “same as before,” or “more than usual.” If the response was “less (quantity) than usual,” the respondent was then asked about the reasons for reduced fish consumption in total.

### Construction of dichotomous study variables

For data analysis, we used dichotomous variables, re-categorized as follows: fish consumption in total quantity (0 = less than pre-COVID, 1 = same/more than pre-COVID); variety (different species) of fish consumption (0 = less than pre-COVID; 1 = same/more than pre-COVID); quality of fish consumption (0 = lower quality than pre-COVID; 1 = same/higher quality than pre-COVID); food purchase (0 = less than pre-COVID; 1 = same/more than pre-COVID); food access (0 = more difficult than usual, 1 = same/easier than pre-COVID). To represent affordability, we used food price (0 = lower/same as pre-COVID, 1 = higher than pre-COVID).

### Hypotheses of the pathways

**Figure 1:**
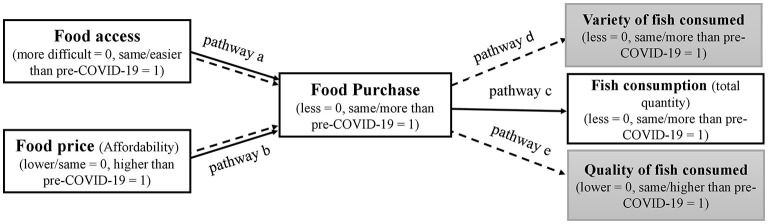
Diagram showing hypothesized basic model (solid arrows, pathway a, b, and c) and extended model (dashed arrows, pathway a, b, d, and e).

Based on the global food environment framework, many potential pathways exist through which food environment may affect diet quality and nutrition outcomes (15, 33). We identified three main pathways: improving food access for increasing purchase (Food Access - Purchase pathway), stabilizing food prices (improving affordability) to increase purchase (Food Affordability - Purchase pathway) and increase in food purchase for increasing consuming nutritious foods (Purchase - Consumption pathway). The Food Access - Purchase pathway assumes that improving access to food will increase the purchase and thus consumption of nutritious foods and add to the diversity and quality of the household diet (15). We posited at first that changes in food access and affordability influenced food purchase and food consumption, particularly that of fish (basic model). If the basic model was accepted, we tested whether changes in food purchase were associated with variety and quality of fish consumed (extended model). Our hypotheses were as follows, and presented in Figure 1:

Hypothesis 1: More difficult food access and higher food price (affordability) will be associated with less food purchase. Furthermore, less food purchase will be associated with less quantity of fish consumption (Basic model).

Hypothesis 2: More difficult food access and higher food price (affordability) will be associated with less food purchase. Furthermore, less food purchase will be associated with a lower variety and lower quality of fish consumption (Extended model).

### Data analysis

Frequencies and percentages were calculated for all categorical variables and means and standard deviations (*SD*) were presented for continuous variables. Phi Coefficient analysis was applied to test correlations among all study variables as they were dichotomous.

A path analysis was employed to test our hypothesized models. We used the estimation method- weighted least square with mean and variance adjustment (WLSMV), the most suitable method if the model contains multiple binary or ordered exogenous or endogenous categorical variables (34–36). Pathway analysis is an extension of multiple regression that enables testing pathways for complicated models with simultaneous estimation of parameters (37). In the basic model, first two exogenous variables “food access” and “food price (affordability)” were modeled with the first endogenous variable “food purchase” and “food purchase” with the second endogenous variable “fish consumption (quantity).” In the extended model, instead of “fish consumption,” both “variety of fish consumption,” and “quality of fish consumption” were modeled as a result of “food purchase.” Both the basic and extended models were adjusted for age, sex, years of schooling and household crowding index. In both models, direct paths from “food access” and “food price” to “fish consumption” were also checked.

Criteria for an acceptable model fit included a non-significant *p*-value of Chi-square (χ^2^), a comparative fit index (CFI), and Tucker-Lewis index (TLI) value >0.90 and root mean square error of approximation (RMSEA) <0.08, and Standardized Root Mean Square Residual (SRMR) <0.08 (38).

We used IBM SPSS Statistics (version 25.0) for descriptive statistics, and R Studio (version 4. 1. 0) with lavaan package (version 0.6–10; Y. Rosseel) for path analysis (34). Standardized path coefficients, *p* values for pathways, and explained variation for endogenous variables (*R*^2^) are reported. Statistical significance was considered if *p* value < 0.05.

## Results

### Characteristics of respondents

A total of 586 adults, including 288 females (49%) and 298 males (51%) participated in this study. The average age of participants was 36 years (*SD* = 12), with 71% being between 25 and 49 years (Table 1). About one-third of the study population (31%) had no formal education, while 13% had 10 years or more of schooling. Thirty-seven percent of respondents were unemployed and 22% were daily wage-earners at the time of the survey. Fifty-nine percent of respondents were living within a household of more than three members per room. Thus, the study population was homogeneous in terms of equal representation of gender, occupation and household characteristics. Ninety-four percent of the respondents reported that COVID-19 had a negative effect on their employment (Table 2), with 47% reporting a total loss of job, 37% reporting a wage drop, and 17% indicating a business economic loss (data not shown).

**Table 1:** Characteristics of the low-income urban households (*n* = 586) during the first wave of the COVID-19 pandemic in Bangladesh.

**Variables**	**Categories**	**% (*n*)**
Age (years)	18–24	14.7 (86)
	25–49	70.6 (414)
	>50	14.7 (86)
Gender	Female	49.1 (288)
	Male	50.9 (298)
Area of residence		
(informal		
settlements)	Tongi	34.8 (204)
	Korail	33.8 (198)
	Mirpur	18.6 (109)
	Dholpur	6.5 (38)
	Shayampur	6.3 (37)
Years of		
schooling	None	30.9 (181)
	1–5	33.1 (194)
	6–9	23.2 (136)
	≥10	12.8 (75)
Current		
occupation	Unemployed	36.9 (216)
	Day laborer	22.2 (130)
	Self-employed	21.3 (125)
	Service and Garment workers	19.6 (115)
Relationship with		
Household Head (HH)	Household Head (HH)	51.5 (302)
	Wife of HH	33.6 (197)
	Child /Parent of HH	10.9 (64)
	Relative of HH	3.9 (23)
Household crowding		
index (HCI)^*^	<3 members per room	41.0 (240)
	≥3 members per room	59.0 (346)

* HCI measured as number of persons per room.

**Table 2:** Changes in the food environment, food purchase, and fish consumption among the low-income urban households (*n* = 586) during the first wave of the COVID-19 pandemic in Bangladesh.

**Domains of the food environment**	**Variables**	**Categories**	**% (*n*)**
Food Acces**s**	How easy was accessing food during Corona?	More difficult than before	89.2 (523)
		Same /easier than before	10.8 (63)
	If difficult, why?^*^	Food was not available in the market	9.6 (56)
		Could not go to market due to restriction	38.9 (228)
		Could not afford	80.2 (470)
		Was in quarantine and other reasons	1.7 (10)
Affordability	What happened to the price of foods during		
	Corona?	Same /decreased than before	16.1 (94)
		Increased than before	83.9 (492)
	Did employment affect due to COVID-19?	Yes	93.7 (549)
Food purchase	How did household food purchase change		
	due to corona?	Less than usual	87.4 (512)
		Same /more than usual	12.6 (74)
	If bought less, why?^*^	Cannot afford to buy more foods	67.7 (397)
		Increased food prices	18.3 (107)
		Cannot go to the market	1.4 (8)
	Most food purchase locations during Corona	Street vendors	31.6 (185)
		Community wet markets/bazar	65.7 (385)
		Food aid/Friends and relatives/Grown own food	2.7 (16)
	Most food purchase locations before Corona^*^	Street vendors	62.6 (367)
		Community wet markets/bazar	95.4 (559)
		Food aid/Friends and relatives/Grown own food	2.6 (15)
(Fish) consumption	How did household fish consumption (total		
	quantity) change during Corona?	Less than pre-COVID	85.8 (503)
		Same / greater than pre-COVID	14.2 (83)
	How did variety (different species) of fish		
	fish consumption change during Corona?	Lower than pre-COVID	86.0 (504)
		Same /more than pre-COVID	14.0 (82)
	How did quality of fish consumption change		
	during Corona?	Lower quality than pre-COVID	47.8 (280)
		Same /higher quality than pre-COVID	52.2 (306)

* Multiple response.

### Impact of the COVID-19 pandemic on the food environment and food purchase

During the COVID-19 pandemic, several changes in the food environment of the urban low-income population in informal settlements were observed, particularly in terms of food access, affordability, and household food purchase (Table 2). Eighty-nine percent of the population reported experiencing difficulty accessing food in general, 39% of the households were unable to visit the market due to mobility restrictions, 80% said they could not afford to buy food in general, and 10% mentioned food was unavailable in the market.

About 84% of the households reported an increase in food prices (Table 2). Furthermore, 87% of the households purchased less food than pre-COVID. As reasons for reduced household food purchase, 78% said they could not afford to buy more foods and 21% mentioned food prices were higher than pre-COVID-19. The most common locations for food purchase were community markets/bazars (66%) and street vendors (32%), both of which decreased from 95 and 63%, respectively, compared to the pre-COVID period.

**Figure 2:**
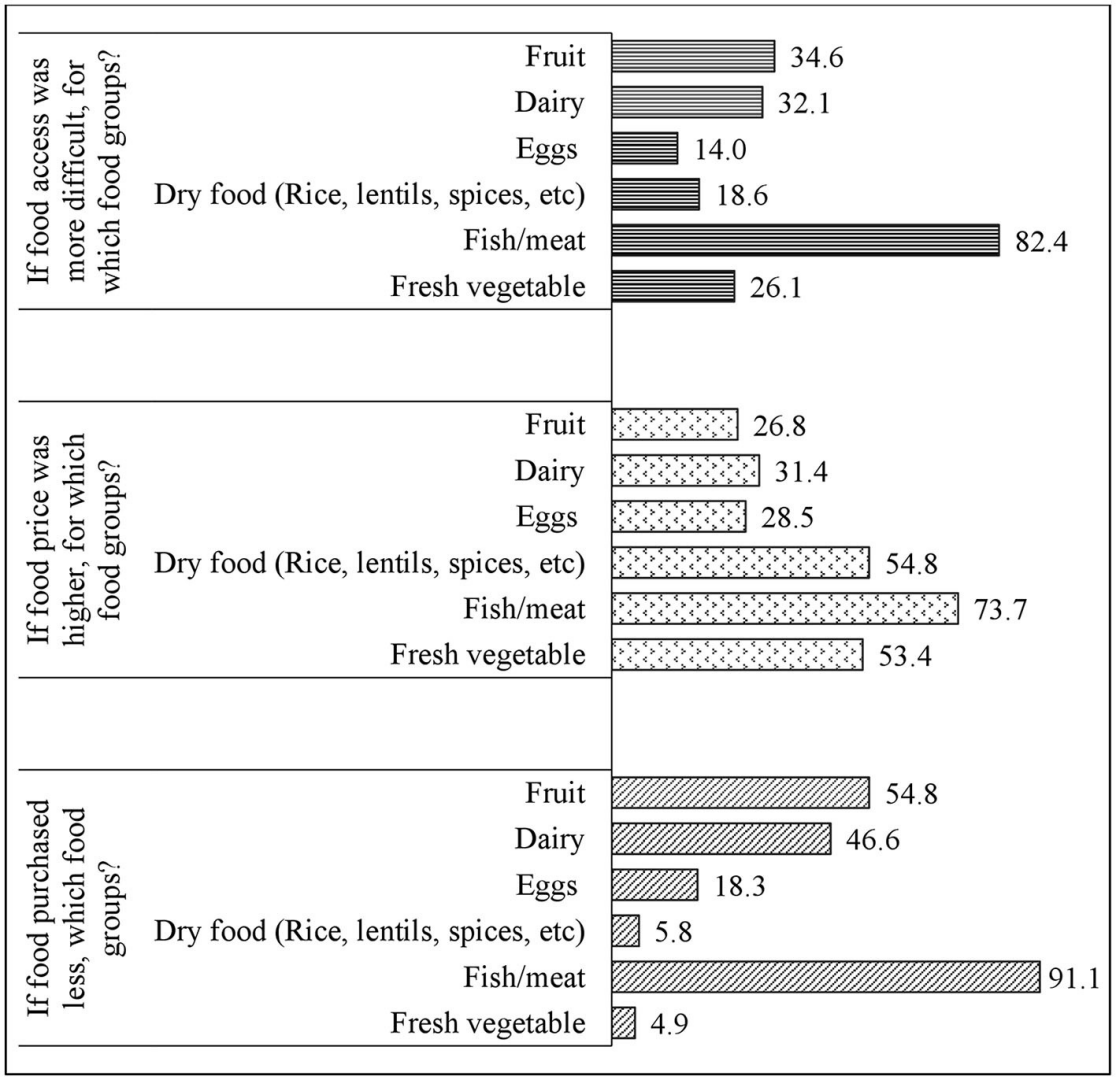
Percentages of respondents reported changes (yes) by food groups when food access was more difficult, price was higher, and purchase was less during the COVID-19 pandemic compared to pre-COVID (*n* = 586, multiple response).

During the COVID-19 pandemic, out of the six main food groups fish and meat were more difficult to access (82% of households), more expensive (74% of households) and purchased less (91% of households) (Figure 2). Fresh vegetables, dry foods, and eggs were the least affected by the COVID-19 pandemic, with 5, 6, and 18% of households reporting less purchases, respectively.

### Impact of the COVID-19 pandemic on fish consumption among the low-income Urban residents

During the first wave of COVID-19 pandemic household fish consumption reduced in quantity, variety (different species) and quality (Table 2). Compared to the pre-COVID period, total fish consumption was reduced (86% of respondents) during the COVID-19 pandemic. The majority of respondents (91%) stated that fish was too expensive and they could not afford to purchase it, while 5% said they could not go to market due to mobility restrictions, and only 2% mentioned that fish was not available in the market (data not shown). Furthermore, the respondents also mentioned that the variety (86% of respondents) and the quality (48% of respondents) of fish consumed was lower than in the pre-COVID period.

### Correlations between the study variables

Food access was positively correlated with household food purchase (phi 0.35, *p* < 0.001) and quantity, variety and quality of fish consumption (phi 0.21–0.26, *p* < 0.001) (Table 3). Fish quality was negatively correlated with food prices (phi −0.22, *p* < 0.001). Food price was negatively correlated with food access (phi −0.19, *p* < 0.001).

**Table 3:** Intercorrelations between the study variables.

**Variables^†^**	**Food access**	**Food price**	**Food purchase**	**Quantity of fish consumption**	**Variety of fish consumption**	**Quality of fish consumption**
Food access	1					
Food price	−0.194^**^	1				
Food purchase	0.349^**^	−0.016	1			
Quantity of fish consumption	0.238^**^	−0.022	0.553^**^	1		
Variety of fish consumption	0.257^**^	0.002	0.557^**^	0.753^**^	1	
Quality of fish consumption	0.211^**^	−0.223^**^	0.240^**^	0.291^**^	0.337^**^	1

** Correlation is significant at the 0.01 level (2-tailed), Phi from crosstab.

† All variables are dichotomous. Food access (0=more difficult, 1=same/easier than usual), Food price (0=lower/same, 1=higher than usual), Food purchase (0=less, 1= same/more than usual), Quantity of fish consumption (0=less, 1=same/greater than usual), Variety of fish consumption (0= less, 1=same/more than usual), Quality of fish consumption (0=lower, 1=same/higher quality than usual).

### Results from path analysis

**Figure 3:**
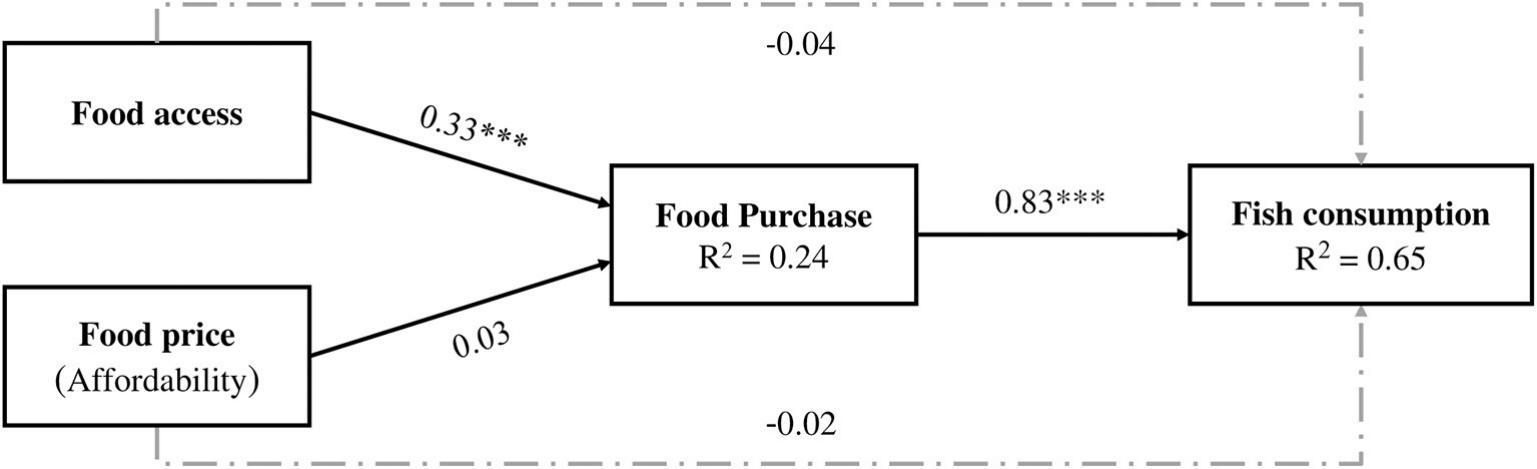
Basic model with standardized coefficients (asterisk (*) showing statistically significance at *p* < 0.001). Good fits of the model indicated by Chi-square = 0.357 (*df* = 2), *p* = 0.837, Comparative Fit Index (CFI) 1.000, Tucker-Lewis Index (TLI) 1.003, Root Mean Square Error of Approximation (RMSEA) 0.000, 90% CI (0.000, 0.047), Standardized Root Mean Square Residual (SRMR) 0.004. Model adjusted for age, gender, education, and HCI. Long dash dotted lines show the direct pathways.

**Figure 4:**
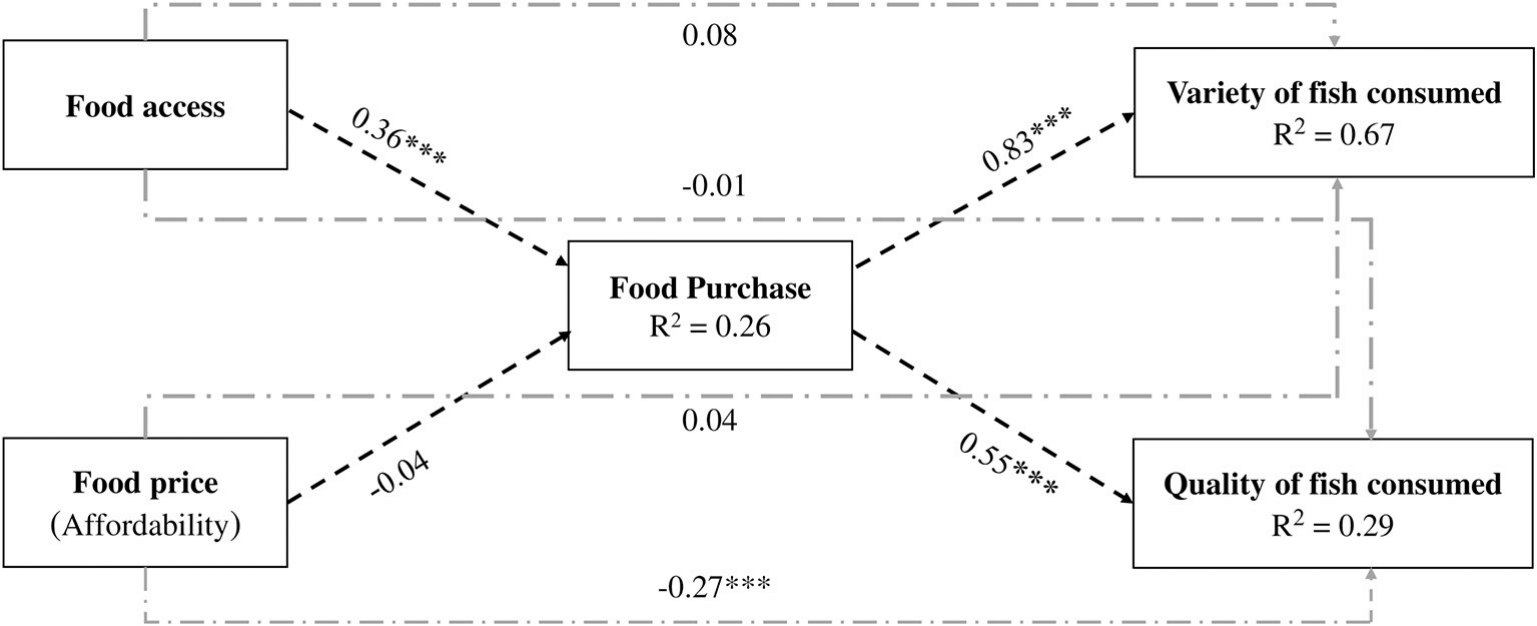
Extended model with standardized coefficients (asterisk (*) showing statistically significance at *p* < 0.001). Reasonable fits of the model indicated by Chi-square = 22.590 (*df* = 4), *p* < 0.001, Comparative Fit Index (CFI) 0.954, Tucker-Lewis Index (TLI) 0.965, Root Mean Square Error of Approximation (RMSEA) 0.089, 90% CI (0.056, 0.129), Standardized Root Mean Square Residual (SRMR) 0.021. Model adjusted for age, gender, education, and HCI. Long dash dotted line shows the direct pathways.

The associations of food access with food purchase, food price with food purchase, food purchase with consumption of fish were examined using path analyses (Figures 3, 4), after adjusting for age, gender, years of schooling and household crowding index (Supplementary Table 1). Both the hypothetical basic model and extended model had acceptable fit indices, indicating that theoretical models were supported by the observed data. In the basic model, food access was positively associated with household food purchase (*b* = 0.33, *p* < 0.001) while food price (affordability) was not. Food purchase was positively associated with consumption of fish (*b* = 0.83, *p* < 0.001). When variety and quality of fish consumption were considered in the extended model, food purchase was positively associated with variety (*b* = 0.83, *p* < 0.001) and quality (*b* = 0.55, *p* < 0.001) of fish consumed. In terms of direct pathways, neither food access nor food price was directly associated with total fish consumption in the basic model. In the extended model, only food price was negatively associated with quality of fish consumption (*b* = −0.27, *p* < 0.001).

## Discussion

This study explored the changes in the personal domains of food environment among low-income residents of Dhaka city during the early months of the COVID-19 pandemic. Moreover, we examined the pathways starting from food access and affordability toward food purchase and consumption, in particular of fish. The food environment was disrupted greatly during the early months of the COVID-19 pandemic in LMICs, including Bangladesh. Previously, several studies documented the negative impact of COVID-19 on household food security and related coping strategies, diet, and nutrition, and these researchers projected possible impacts of the COVID-19 on the food environment based on different theoretical conceptual frameworks (1, 2, 18, 20, 29, 39, 40). However, to the best of our knowledge, the contribution of food access and food prices (affordability) to food purchase and fish consumption has not been reported before. Also the pathways within the global food environment framework have not yet been evaluated using real-life data from a survey conducted during the early stage of the COVID-19 pandemic among households in informal settlements in Dhaka city of Bangladesh.

Our findings showed that 89% of the households in informal settlements experienced difficulties in accessing food during the COVID-19 pandemic. A recent systematic review on the impact of the COVID-19 pandemic on diet quality, food, and nutrition security in LMICs described similar findings about difficulties in food access among the lowest-income quintile at the beginning of the COVID-19 pandemic as well as after lifting the lockdown (1). In our study, the proportion of households (89%) experiencing difficulty in accessing food was higher than in another study in both the urban and rural population of Bangladesh, which reported lower access to the same amount (45%) and same types of foods (61%) during the pandemic compared to the pre-COVID period (7). However, these studies did not focus on the urban low-income population in informal settlements (7, 29). The high reports of difficulty in accessing food among the urban low-income population indicates their vulnerability to crises such as the COVID-19 pandemic that disrupted urban food environment.

We found that in terms of foods groups, fish and meat were the most difficult to access (82% of respondents faced difficulty), the most expensive (74% of respondents reported high price) and the least purchased (91% of respondents reported purchasing less) during the pandemic. Similar findings regarding increased food insecurity and eating only “potato and vegetables” with rice were observed among the low-income urban and rural population in Bangladesh during the COVID-19 pandemic (5, 8, 41). The restrictions on transportation during the pandemic disrupted the supply chain and this in turn probably limited availability and increased the price of fish and meat as described in a previous study in Bangladesh (20). In a different study, researchers reported that local government took some measures that consequently stabilized the prices of essential foods after the lockdown (3). These measures could explain our observation of increased purchase of dry items: rice, lentils, as well as eggs, and vegetables. However, it is ambiguous whether fish and meat were considered as essential foods and whether any price control measures were taken for this food group. The increased price of fish and meat and the economic crisis probably forced the people to buy dry food items (rice, lentils, onion, potatoes, flours) which were cheaper to meet their hunger and move away from nutritious animal-sourced foods, particularly fish (4, 5, 30).

We found that food access was positively associated with food purchase, but food price was not. Previous findings reported food insecurity during the pandemic (42). It is plausible that difficulties in food access during the pandemic might have been mainly through loss of job and reduced family income, reducing the ability to buy food among low-income consumers (27, 42, 43). Furthermore, we observed that restrictions on public mobility (39% of households) and unavailability of food (10% of households) contributed to limited access to food and thus reduced food purchases. Apart from these, supply chain disruptions, restriction of the mobility of vendors, limited opening hours of retailers, and market displacement observed in LMICs, including Bangladesh, could have contributed to limited access to food for the study population (29, 31, 40, 44–46). This suggests that, rather than high food prices, limited food access during the pandemic may have lowered food purchase.

Our analysis showed that during the pandemic when food purchase was reduced, the quantity, variety and quality of fish consumed was negatively affected. Similar findings about reduced grocery shopping and decreased frequency and amount of fish consumption was observed among low-income and middle-income residents of Dhaka during the early days of pandemic (4). In terms of reduced variety of fish eaten, researchers have reported the tendency of low-income consumers from Asia and Africa to purchase less expensive fish species, such as carps (*Cyprinus* species*)*, tilapia (*Oreochromis* species*)*, pangas (*Pangasius)* (47). Studies from Dhaka city and other cities of Bangladesh reported that some of these low cost fish were not available in the market and were pricier during the pandemic (4, 8). Our findings on the perceived reduction in the quality of fish consumed warrant some discussion. The perception of low quality of fish consumed reported by the respondents may mean that the fish was not fresh, had an unpleasant smell, and were unappealing (48). Our observation suggested that during the pandemic, urban low-income residents often obtained soft and slightly rotten fish at the end of the day when vendors sold them cheap or give them away. It was interesting that increased price only affected the quality of fish but not the quantity or variety. It is possible that since fish is the most commonly consumed animal sourced food and an integral part of the Bangladeshi diet (10, 11), urban low-income residents compromised the quality of fish instead of reducing the intake in quantity or variety. However, further research is necessary to understand what the lower quality of fish consumption meant in terms of food safety concerns of the low-income consumers and how they coped with such concerns.

An important limitation of the present study is its cross-sectional design, which does not allow conclusions on causality of the associations. While it is plausible that food access was associated with food purchase, and that food purchase was associated with fish consumption, food access was also not directly associated with overall fish intake, as well as the variety and quality of fish consumed. We anticipate that low-income populations, particularly residents in informal settlements, were less likely to panic-buy, hoard, or store fish at home and were unable to eat fish outdoors due to being severe food insecure during the pandemic compared to rural households in Bangladesh (49). Secondly, our data were collected through self-report and were subject to potential social desirability bias. However, we interviewed over the phone, which may reduce social desirability bias compared to face-to-face interviews (50). Thirdly, we did not assess food or fish intake in frequency or quantity (e.g., the number of times or grams) or the number of fish species which could have enriched our findings. Hence, our cross-sectional study may serve as a prequel to further longitudinal designs to confirm the causal relationship between food access and affordability with food purchase and fish consumption.

Our study has several strengths, including a large sample size that increased study power, as well as the use of PPS sampling technique to enroll households, resulting in a more representative study sample of low-income households in Dhaka city, Bangladesh. We used structure equation modeling for path analysis to test theoretical pathways based on the global food environment framework of Turner et al. (15). Pathway analysis is an extension of multiple regression that provides estimates of the magnitude and significance of hypothesized causal connections among sets of variables displayed through the use of path diagrams (37). In comparison to earlier research in 119 countries, including Bangladesh (29), to the best of our knowledge this study was the first to assess the food environment among low-income urban residents in informal settlements of Dhaka city, who were most vulnerable during the first phase of the COVID-19 pandemic.

## Conclusion

The findings of this study demonstrate that the COVID-19 pandemic negatively affected the food environment domains, particularly food access, food prices (affordability), food purchase and consumption of fish among the urban low-income households in Bangladesh. Reduction in food purchase during the COVID-19 pandemic negatively affected the overall fish consumption, the variety, and quality. Food access was positively associated with food purchase and food purchase was associated positively with fish consumption. Food price was associated with quality of fish consumed. This study provides a unique opportunity to offer insights to understand the pathways more explicitly based on the Turner et al. framework (15) and contributes to the literature of the food environment domains (food access and price) and the fish consumption during the early months of the COVID-19 pandemic. Therefore, it is advisable that future policy actions include a more equitable food environment that may alleviate limitations in accessing food, prevent increase in food prices, and improve affordability for urban low-income households during emergencies such as COVID-19. The urban low-income residents in informal settlements should be included in effective social safety net programs. They should have equal access to safe, nutritious foods, such as fish, with sufficient quantity, variety, and quality to ensure that they are well-nourished and healthy.

## Data availability statement

The raw data supporting the conclusions of this article will be made available by the authors, without undue reservation.

## Ethics statement

The study involving human participants was reviewed and approved by Institutional Review Board (IRB) of icddr,b (PR-20075). The participants provided their verbal informed consent through phone calls to participate in this study.

## Author contributions

MA conceptualized the research question, conducted the data analysis, and drafted the manuscript. EF and SR have contributed to the research concept, data analysis, and interpretation. ET and ST contributed to formulating the manuscript, interpreting the results, edited the manuscript, and critically reviewed the initial draft. All authors have seen and approved the final version.

## Funding

Data collection for the study was funded by the Swedish International Development Cooperation Agency (Grant Number-01455). This article was prepared as part of the CGIAR Research Programs: (1) Agriculture for Nutrition and Health (A4NH; Food System for healthier diets flagship-1, https://a4nh.cgiar.org/our-research/flagship-1/) and (2) Fish Agri-Food Systems (FiSH) led by WorldFish.

## Conflict of interest

The authors declare that the research was conducted in the absence of any commercial or financial relationships that could be construed as a potential conflict of interest.

## Publisher's note

All claims expressed in this article are solely those of the authors and do not necessarily represent those of their affiliated organizations, or those of the publisher, the editors and the reviewers. Any product that may be evaluated in this article, or claim that may be made by its manufacturer, is not guaranteed or endorsed by the publisher.
