# Supplementary material for: How did the COVID-19 pandemic affect food environment, food purchase, and fish consumption among low-income urban households in Bangladesh—A path analysis
Source: Front Public Health. 2022 Sep 15;10:994236. doi: 10.3389/fpubh.2022.994236 (PMC9521681; doi:10.3389/fpubh.2022.994236)
Supplement: Supplementary file 1 [file Table_1.docx]

Supplementary Material

# Supplementary Table

Supplementary Table 1: Path coefficients of the basic and extended model †

| **Basic model** | **X** | **→** | **Y** | **B** | **95% CI** | **S.E.** | **Z -value** | ***p*** | ***b*** | |
| --- | --- | --- | --- | --- | --- | --- | --- | --- | --- | --- |
| pathway a | Food access | → | Food purchase | 1.232 | (0.851, 1.613) | 0.194 | 6.34 | < 0.001 | 0.33 | |
| pathway b | Food prices | → | Food purchase | 0.088 | (-0.274, 0.451) | 0.185 | 0.48 | 0.63 | 0.028 | |
| pathway c | Food purchase | → | total fish consumption | 0.777 | (0.684, 0.870) | 0.047 | 16.38 | < 0.001 | 0.83 | |
|  |  |  |  |  |  |  |  |  |  | |
| **Extended model** | **X** | **→** | **Y** | **B** | **95% CI** | **S.E.** | **Z -value** | ***p*** | ***b*** | |
| pathway a | Food access | → | Food purchase | 1.331 | (0.935, 1.727) | 0.202 | 6.586 | < 0.001 | 0.355 | |
| pathway b | Food prices | → | Food purchase | -0.111 | (-0.440, 0.219) | 0.168 | -0.66 | 0.51 | -0.035 | |
| pathway d | Food purchase | → | Variety of fish consumption | 0.779 | (0.648, 0.873) | 0.048 | 16.16 | < 0.001 | 0.829 | |
| pathway e | Food purchase | → | Quality of fish consumption | 0.049 | (0.358, 0.622) | 0.067 | 7.26 | < 0.001 | 0.552 | |
| Abbreviation: B: unstandardized coefficient. CI: Confidence Interval, S.E.: standard error, *b*: the standardized path coefficient.  †Each model adjusted for age, gender, years of schooling and household crowing index  → showing direction of the model | | | | | | | | | |  |
